# Effects of K‐12 School District Nonpharmaceutical Interventions on Community‐Level Prevalence of Acute Respiratory Infection During the COVID‐19 Pandemic

**DOI:** 10.1111/irv.70139

**Published:** 2025-07-13

**Authors:** C. He, M. D. Goss, D. Norton, G. Chen, A. Uzicanin, J. L. Temte

**Affiliations:** ^1^ Department of Family Medicine and Community Health University of Wisconsin Madison Wisconsin USA; ^2^ Department of Biostatistics and Medical Informatics University of Wisconsin Madison Wisconsin USA; ^3^ Centers for Disease Control and Prevention Atlanta Georgia USA

**Keywords:** acute respiratory infection, COVID‐19, K‐12 schools, nonpharmaceutical interventions, public health surveillance, respiratory virus transmission, SARS‐CoV‐2

## Abstract

**Background:**

Responding to the COVID‐19 pandemic, kindergarten through 12th grade schools implemented nonpharmaceutical interventions (NPIs). The effects of school‐based NPIs on broader community levels of acute respiratory infection (ARI) have not been defined. We utilized an existing longitudinal cohort of households reporting weekly ARI cases to evaluate the effects of evolving school districtwide NPIs on ARI activity at eight transition points from December 2019 through October 2022.

**Methods:**

Household ARI data were reported through the GReat Oregon Child Absenteeism due to Respiratory Disease Study (ORCHARDS) Vaccine Effectiveness Study—a prospective cohort study based in the Oregon School District (OSD) (GROVES). Participating GROVES families completed weekly online surveys with respiratory illness updates. Mixed effects logistic regression was used to examine the association between eight school‐related transition events during the COVID‐19 pandemic and changes in the trajectory of ARI risk for GROVES family members, while accounting for family clusters. Transition events were assessed using a ±4‐week window of community data.

**Results:**

Opening schools with maximal NPIs (mandated masking and physical distancing, with hybrid education) was not associated with increased community ARI activity. The four transition events associated with significant ARI risk trajectory increases included summer breaks (June 2020, *p* = 0.001; June 2021, *p* = 0.002), and the start of school with mandatory masking only (September 2021, *p* < 0.001) or without NPIs (September 2022, *p* < 0.001).

**Conclusions:**

School‐based NPI implementation was associated with reduced risks for community ARI activity. Enhanced surveillance platforms such as the weekly online surveys used in this study are valuable tools for better understanding and monitoring SARS‐CoV‐2 and respiratory virus transmission in schools and surrounding communities.

## 1 Introduction

In pandemic preparedness and responses that predated the emergence of severe acute respiratory syndrome coronavirus 2 (SARS‐CoV‐2), use of nonpharmaceutical interventions (NPI) has been recommended to limit pathogen transmission and reduce morbidity, mortality, and overloading of the healthcare system from the earliest stages of an evolving pandemic, when vaccines and other pharmaceutical preventive and treatment options are often not available [1, 2]. Initial NPI measures designed to slow the spread of SARS‐CoV‐2 included closure of schools, businesses, and public transport; physical distancing; public event and gathering bans; and the use of face masks in public areas.

Kindergarten through 12th grade (K‐12) schools—tasked with maintaining their educational missions—employed multiple NPIs throughout the coronavirus disease 2019 (COVID‐19) pandemic. In the United States, these included preemptive school closures (most with virtual school options) [3], hybrid education (e.g., split classrooms receiving alternating in‐person and virtual education), physical distancing, and mandatory/voluntary use of face masks [3, 4]. Moreover, K‐12 schools transitioned across interventions as the pandemic unfolded [4].

School‐aged children have been recognized as drivers of respiratory virus transmission due to prolonged congregation within schools, prolonged viral shedding, and larger social networks [5, 6, 7]. School reopening dates have preceded local waves of pandemic influenza A(H1N1) during the fall of 2009 [8], and during nonpandemic years, increases in medically attended ARI for individuals ≤ 65 years have occurred shortly after the beginning of the academic year [9], suggesting that school session status may impact community ARI levels. For this reason, it could be hypothesized that if effective NPIs are deployed at appropriate times in K‐12 school settings, they might affect subsequent prevalence of ARI across the broader community. Although NPI use within schools limited rates of SARS‐CoV‐2 infection and in‐school transmission when physical distancing and masking strategies were enforced [10], little evidence exists for the extension of this effect beyond the school setting.

In this study, we utilized a prospective community‐based ARI surveillance program—established in November 2019—to assess the potential effects of NPIs in K‐12 school settings on community‐level prevalence of ARI during the COVID‐19 pandemic. We specifically focused on eight transition points based on the timing of NPIs employed and the school calendar.

## 2 Methods

### 2.1 Location

We worked with the Oregon School District (OSD: Dane County, South Central Wisconsin). We have a long‐standing relationship with the OSD through the Oregon Child Absenteeism due to Respiratory Disease Study (ORCHARDS), which has been in place since 2013 [11, 12]. The OSD provides education to over 4000 K‐12 students across seven schools. The total population enclosed by the OSD boundaries is approximately 22,000 people [11]. ORCHARDS‐generated weekly data on ARIs in participating K‐12 students with laboratory‐confirmed etiology, used to contextualize the data from the present study, were previously published [13].

### 2.2 Timeframe

**FIGURE 1:**
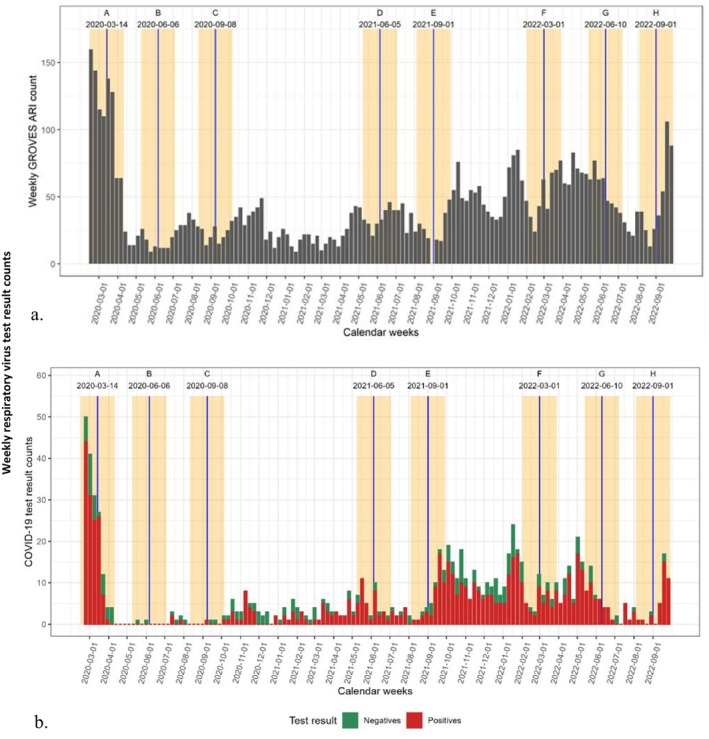
**(a)** Weekly acute respiratory infection (ARI) cases reported among the GReat ORCHARDS Vaccine Effectiveness Study (GROVES) participant families from late November 2019 through September 2022. The eight school‐based transition events are indicated by blue lines, and the ±4‐week period by yellow bars. In chronological order, events are: (A) 3/14/2020: school closure during early COVID‐19 pandemic; (B) 6/6/2020: start of summer break 2020; (C) 9/8/2020: start of school year with mandatory masking and physical distancing plus hybrid education; (D) 6/5/2021: start of summer break 2021; (E) 9/1/2021: start of school year with mandatory masking only; (F) 3/1/2022: change from mandatory to optional use of face masks in schools; (G) 6/10/2022: start of summer break 2022; (H) 9/1/2022: start of school year with no nonpharmaceutical intervention (NPI) requirements. **(b)** Weekly ARI cases in persons recruited for participation in the ORegon CHild Absenteeism due to Respiratory Disease Study (ORCHARDS) from late November 2019 through September 2022. The eight school‐based transition events are indicated as above. Red bars represent participants from whom specimens had a positive respiratory virus* identification; green bars represent participants from whom specimens had negative respiratory virus identification. *Respiratory viruses included: influenza (Flu) AH1, FluAH3, FluB, respiratory syncytial virus (RSV) A, RSV B, coronavirus (CoV) 229E, CoV OC43, CoV NL63, CoV HKU1, human metapneumovirus, rhinovirus/enterovirus, adenovirus, parainfluenza virus (PIV) 1, PIV‐2, PIV‐3, PIV‐4, human bocavirus, and severe acute respiratory syndrome coronavirus 2 (SARS‐CoV‐2).

We evaluated the period from December 2019 through October 2022. This period encompassed a significant influenza outbreak (2019/2020: influenza A[H1N1] and influenza B), the emergence of SARS‐CoV‐2 and its subsequent variants, implementation of NPIs within the OSD, and the removal of all NPIs at the start of the 2022–2023 academic year. We focused on eight specific transition points in the study period. Transition events were defined as discrete points in time that had the potential to change the trajectory of respiratory infection risk in the broader school community (i.e., the start or end of the school year and NPI initiation or cessation), and are shown longitudinally in Figure 1a. ARI activity during the same timeframe is shown in Figure 1b.

The OSD ended in‐person instruction for the 2019–2020 school year on **March 13, 2020 (Event 1)**, in advance of the Safer at Home Emergency Order (#12), set forth by the Wisconsin Department of Health Services, which included the shutdown of all schools, nonessential businesses, and activities; physical distancing; and prohibition of public gatherings and nonessential travel [14, 15]. Virtual schooling remained in place through **June 6, 2020 (Event 2)**, which marked the beginning of summer break. OSD students returned to school on **September 8, 2020 (Event 3)** with a phased reopening by grade level, beginning September 2020 (Grades K–2) and continuing through January 2021 (Grades 3–6) and February 2021 (Grades 7–12). Face masks and physical distancing were enforced for students attending in‐person classes, and the option to continue full virtual learning was offered for all OSD grade levels during the 2020–2021 school year (~25% of students opted to continue virtual education). Morning or afternoon block scheduling was also implemented, thus reducing the student density while in class and the size of student gatherings in the cafeteria. All NPIs remained in place through the end of the school year on **June 10, 2021 (Event 4)**. OSD students returned to fully in‐person school with mandatory face masking on **September 1, 2021 (Event 5)**. The face covering emergency order for Dane County public schools ended on **March 1, 2022 (Event 6)**. Summer break began on **June 10, 2022 (Event 7)**. OSD students returned to school without any NPI measures in place on **September 1, 2022 (Event 8)**.

### 2.3 Outcome Data

We used household ARI events as reported to the GReat ORCHARDS Vaccine Effectiveness Study ([GROVES]—a prospective cohort study based in the OSD). GROVES was initially envisioned as a 1‐year prospective cohort study designed to collect household data on ARIs for use in assessing influenza vaccine effectiveness. Beginning in the fall of 2019, families with school‐aged children who [1] had previously participated in ORCHARDS and [2] consented to be notified for future studies were contacted via email with an invitation to enroll in GROVES. Families completing the GROVES e‐consent and screening survey were enrolled. Basic demographic information was collected, including school attendance, work environment, and intention to receive the seasonal influenza vaccine for that year. Enrolled families were asked to respond to weekly electronic prompts (delivered by email) and complete a weekly online survey (supplement) with respiratory illness updates.

We used the weekly survey instrument to collect demographic, epidemiologic, symptom information, and recent travel history for any family members with respiratory symptom onset (ARI) in the past 7 days, or any positive influenza (starting in December 2019) or SARS‐CoV‐2 (added in October 2020) test results for family members in the past week. Testing could have been performed by public health, a clinical facility, using an at‐home test, or through ORCHARDS participation. The weekly proportion of participating families reporting one or more household members with new ARI symptoms (Weekly % ARI) was used to monitor ARI activity in the cohort and the school community. Study data and surveys were collected and managed using REDCap electronic data capture tools [16] hosted at the University of Wisconsin‐Madison. All components of the study were reviewed and approved by the University of Wisconsin Health Sciences Institutional Review Boards (protocol 2013–1357).

### 2.4 Statistical Analysis of Transition Events

Mixed effects logistic regression was used to examine the association between the date of eight school‐related transition events during the COVID‐19 pandemic and changes in the trajectory of ARI risk for the GROVES family members, while accounting for family clusters. Separate regression models with the same structure were run for each transition, the outcome being weekly ARI number as reported by GROVES families, paired with the remaining number of family members without an ARI.

A ±4‐week window of data was used from GROVES around the week that the transition occurred. Two longitudinal time covariates were included in each model: a count of the weeks centered around the week of the transition (e.g., weeks −4 through Week 4, with Week 0 being the transition event week), and the “time after transition” covariates, which is a value of 0 for all weeks before the transition, and equal to the “week count” after the week of the transition. This created a piecewise linear model with respect to time, allowing a “kink” in the ARI risk trajectory at the week of the transition while forcing continuity in the ARI trajectory at the transition week, and allowing slopes before and after the transition week to be different. Thus, the time‐after‐transition covariate is the change in the ARI slope after the transition event.

Other covariates in the models included the number of household members per bedroom for each family, the number of adults working outside of the house, the number of children attending school in person, and a weekly measure of ARI in the school community. This weekly ARI community measure was created from the ORCHARDS dataset, from which GROVES families were recruited, and represented the number of recruited ORCHARDS participants meeting ARI symptom criteria [11] for the corresponding week of GROVES data. ORCHARDS ARI counts are reported as raw counts. This item was rescaled to be a proportion of the maximum weekly ORCHARDS ARI value that occurred (i.e., divided by the maximum ORCHARDS ARI value), as some models experienced convergence issues without rescaling. Random effects in the models include family‐specific random intercepts.

No major concerns of overdispersion were found after fitting. The 95% confidence intervals around the fitted regression lines were created using a standard error estimation from parametric bootstraps of the fitted models over 200 iterations at each week and assumed asymptotic normal behavior of the conditional means. All analyses were performed in R version 4.3.1 and utilized the lme4 package for regressions along with the boot package for bootstrapping [17, 18, 19].

To further assess the association between the change in reported weekly ARI and the initial transition event (pandemic‐driven school closure after March 13, 2020) that occurred coincidentally with a rapid decline of influenza cases across Wisconsin [20], a ±16‐week window of data from GROVES was analyzed around this transition week using interrupted time‐series logistic regression. To assess if the slope/intercept interrupt was associated with a change in family illness probability, a likelihood ratio test was conducted versus a model without the interrupt. Slope and intercept change coefficients were individually examined.

## 3 Results

### 3.1 Demographics

Study participants' characteristics are shown in Table 1. There was stability in mean age, sex, and household size across the study period. The proportion of students receiving virtual education declined as in‐person school attendance increased. High levels of work outside of the home were noted across the study periods.

**TABLE 1:** Demographic characteristics of participating families during three academic years of the GROVES* study: 2019–2020, 2020–2021, and 2021–2022.

	Mean (SD)	2019–2020	2020–2021	2021–2022
Family size	4.09 (0.90)	4.14	4.10	4.11
Number of bedrooms	3.61 (0.79)	3.59	3.56	3.52
Household members per bedroom	1.17 (0.30)	1.15	1.15	1.17
Adults per household	2.05 (0.60)	2.06	2.11	2.07
**Race/ethnicity**		2019–2020	2020–2021	2021–2022
White non‐Hispanic		756 (89.6%)	711 (88.9%)	639 (89.2%)
White Hispanic		23 (2.7%)	25 (3.1%)	21 (2.9%)
Other or undisclosed		65 (7.7%)	64 (8.0%)	56 (7.8%)
**Sex**		2019–2020	2020–2021	2021–2022
Male		426 (50.5%)	399 (49.9%)	359 (50.1%)
Female		410 (48.6%)	382 (47.8%)	324 (45.3%)
Sex undisclosed		8 (0.9%)	19 (2.4%)	33 (4.6%)
**Activities outside the household**		2019–2020	2020–2021	2021–2022
Children enrolled in school	1.57 (0.95)	407	349 (89.7%)	322 (90.7%)
Children attending virtual school	0.50 (0.90)	**	241 (69.1%)	5 (1.6%)
Children attending school in person	1.24 (1.11)	**	11 (3.2%)	313 (97.2%)
Children attending hybrid school†	0.21 (0.61)	**	97 (27.8%)	4 (1.2%)
Adults working outside of house (parent or other)	1.82 (0.76)	345 (82.1%)	272 (66.2%)	269 (74.5%)
Individuals working outside of house (includes < 18 years or “Child”)		383 (91.2%)	298 (72.5%)	308 (85.3%)
**Families participating during each transition event**	N			
2020‐03‐14: School closure with—Virtual education	204			
2020‐06‐06: Summer break	197			
2020‐09‐08: School with masking/distancing/hybrid ed.	197			
2021‐06‐10: Summer break	193			
2021‐09‐01: School with masking only	128			
2022‐03‐01: School with optional masking	173			
2022‐06‐10: Summer break	168			
2022‐09‐01: School with no requirements	146			

* GROVES = GReat ORCHARDS Vaccine Effectiveness Study.

** data not collected until 2020–2021 school year.

split classrooms receiving alternating in‐person and virtual education.

### 3.2 Adherence to Protocol

Although GROVES was originally structured as a 1‐year study, participating households were rerecruited annually, and overall participation in GROVES varied (Table 1): 204 families (844 individuals) during the 2019–2020 school year, 195 families (800 individuals) for 2020–2021, 174 families (716 individuals) for 2021–2022, and 159 families (642 individuals) for 2022–2023. Weekly survey response rates, however, remained relatively stable across school years, ranging from 78.3% to 100% with an average weekly response rate of 94.1% (standard deviation = 4.1%). GROVES participant families completed weekly surveys each week from November 25, 2019, through October 2, 2022, with the exception of 1 week (August 23–29, 2021), while recruitment for the following school year was being conducted. The proportion of families who reported at least one family member with respiratory symptoms was monitored and recorded as Weekly % ARI and ranged from 2.7% (survey date: January 18, 2021) to 54% (survey date: December 16, 2019) (mean = 17.92%, median = 14.09%).

### 3.3 Trends Across Transition Events

**FIGURE 2:**
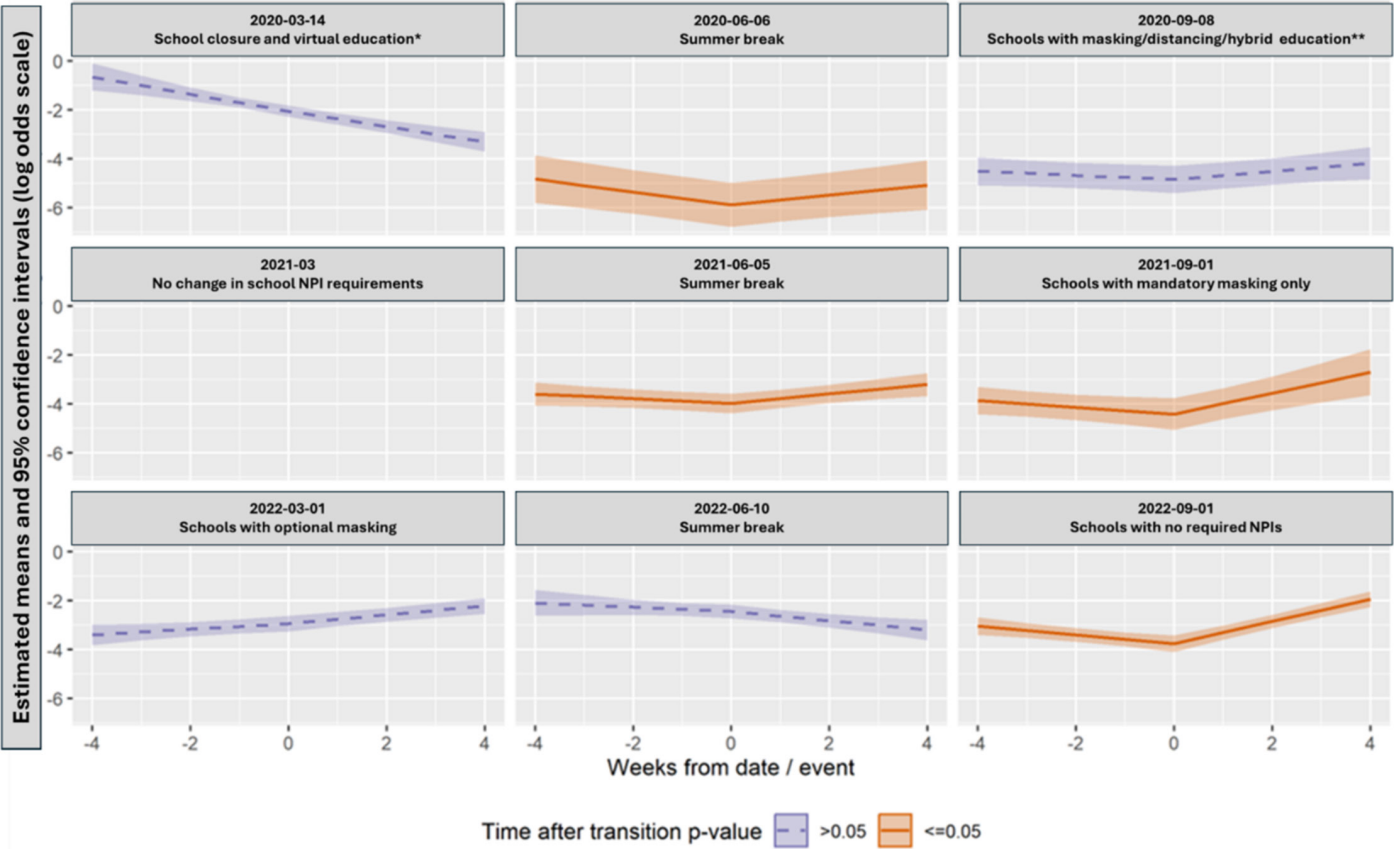
Change in the slope on the logit scale (log‐odds) of acute respiratory infection (i.e., ARI risk trajectory [estimated means—log‐odds scale]) in households with “kink” at transition event date for eight transitions in intensity of nonpharmaceutical interventions (NPIs) in the school district: two in March: 2020 (school closure—reduced transmission risk) and 2022 (change from mandatory masking to optional masking—increased transmission risk), three in June: 2020, 2021 (beginning of summer breaks with increased transmission risk due to the end of school‐based interventions) and 2022 (reduced transmission risk due to less congregation), and three in September: 2020 (beginning of school year with mandatory masking, physical distancing and hybrid [combined in‐person and virtual] education‐reduced transmission risk), 2021 (beginning of school year with mandatory masking only‐reduced transmission risk), and 2022 (beginning of school year no required NPI‐increased transmission risk). There was no transition event in March of 2021; panel added for date alignment. Significant (*p* < 0.05) findings are depicted by orange shading. *all students received virtual education for the remainder of the 2020–2021 academic year. **hybrid education included both in‐person and virtual education with 50% of students receiving in‐person education in the morning and 50% receiving in‐person education in the afternoon.

Similar patterns of ARI incidence were noted among GROVES household members (Figure 1a) and ORCHARDS K–12 participants (Figure 1b). Of the eight transition events examined (Figure 2), four were significantly associated with a change in the ARI risks slope, and all four were estimated to be increases in the risk trajectory: the summer break of June 2020 (β = 0.46, 95% CI 0.18—0.75, *p* = 0.001), the summer break of June 2021 (β = 0.29, 95% CI 0.11—0.47, *p* = 0.002), school starting with required masking in September 2021 (β = 0.57, 95% CI 0.29—0.84, *p* < 0.001), and school starting with no NPI requirements in September 2022 (β = 0.64, 95% CI 0.45—0.82, *p* < 0.001).

Results around time‐after‐transition are reported on the scale of the regression and interpreted as the slope change on the logit scale (log‐odds) of ARI risk, instead of the typical odds ratio (OR) scale, in order to avoid dependence on the reference timepoint and a particular passage of time. The remaining main effect covariates that did not vary by time are reported on the OR scale for better interpretation (Table 2). Several other covariates in regression models were statistically significant: the scaled weekly ORCHARDS ARI count, which was positively associated with ARI risk for two transition models (school closure in March 2020 and a large‐magnitude association in September 2022 for schools opening without restriction); members per bedroom was positively associated with ARI risk for September 2022 when schools opened without restriction; and the number of children attending in‐person school was positively associated with ARI risk for March 2022 when schools moved to optional masking.

**TABLE 2:** Logistic regression results for eight transition events with covariates included in the model. Significant transition events (*p* < 0.05) are highlighted. Events significantly associated with a change in acute respiratory infection (ARI) risk trajectory are significant in regard to *effect of event* (*p* < 0.05). ORCHARDS, ORegon CHild Absenteeism due to Respiratory Disease Study.

Transition/regression model	Covariate	OR estimate	OR 95% CI	*p*
**3/14/2020** School closure with virtual education	Effect of event	1.04	−0.09, 0.17	0.5452
	Week count (time)	0.70	−0.49, −0.21	0.0000
	Members/bedroom*	1.21	−0.32, 0.7	0.4614
	Scaled ORCHARDS weekly ARI	0.13	−2.95, −1.11	0.0000
	Children attending school	1.11	−0.14, 0.35	0.3999
	Adults working outside house	0.82	−0.41, 0.02	0.0731
**6/6/2020** Summer break	Effect of event	1.59	0.18, 0.75	0.0014
	Week count (time)	0.77	−0.41, −0.11	0.0006
	Members/bedroom	0.54	−1.89, 0.64	0.3353
	Scaled ORCHARDS weekly ARI	0.00	−36.85, 8.4	0.2179
	Children attending school	1.54	−0.19, 1.04	0.1717
	Adults working outside house	0.79	−0.78, 0.32	0.4122
**9/8/2020** School with masking/distancing/hybrid education	Effect of event	1.28	0, 0.5	0.0537
	Week count (time)	0.92	−0.21, 0.04	0.1881
	Members/bedroom	0.62	−1.58, 0.62	0.3907
	Scaled ORCHARDS weekly ARI	0.00	−40.54, 11.95	0.2858
	Children attending school	1.53	−0.1, 0.95	0.1107
	Adults working outside house	0.75	−0.77, 0.2	0.2504
**6/10/2021** Summer break	Effect of event	1.34	0.11, 0.47	0.0019
	Week count (time)	0.91	−0.21, 0.02	0.0940
	Members/bedroom	0.89	−0.87, 0.63	0.7549
	Scaled ORCHARDS weekly ARI	3.37	−3.28, 5.71	0.5961
	Children attending school	1.26	−0.21, 0.68	0.3046
	Adults working outside house	1.08	−0.24, 0.39	0.6502
**9/1/2021** School with masking only	Effect of event	1.77	0.3, 0.84	0.0000
	Week count (time)	0.88	−0.31, 0.06	0.1916
	Members/bedroom	1.10	−0.65, 0.83	0.8066
	Scaled ORCHARDS weekly ARI	0.03	−7.74, 0.39	0.0761
	Children attending school	1.32	−0.02, 0.58	0.0717
	Adults working outside house	0.78	−0.58, 0.09	0.1518
**3/1/2022** School with optional masking	Effect of event	1.06	−0.1, 0.23	0.4532
	Week count (time)	1.12	0, 0.23	0.0426
	Members/bedroom	1.02	−0.67, 0.71	0.9577
	Scaled ORCHARDS weekly ARI	0.01	−10.31, 0.4	0.0700
	Children attending school	1.45	0.11, 0.64	0.0060
	Adults working outside house	0.91	−0.34, 0.16	0.4710
**6/10/2022** Summer break	Effect of event	0.90	−0.25, 0.05	0.1976
	Week count (time)	0.92	−0.22, 0.05	0.1962
	Members/bedroom	1.15	−0.45, 0.73	0.6331
	Scaled ORCHARDS weekly ARI	0.07	−7.62, 2.29	0.2922
	Children attending school	0.86	−0.38, 0.08	0.1874
	Adults working outside house	0.91	−0.31, 0.12	0.3777
**9/1/2022** School with no requirements	Effect of event	1.86	0.43, 0.82	0.0000
	Week count (time)	0.85	−0.29, −0.04	0.0088
	Members/bedroom	3.16	0.43, 1.87	0.0017
	Scaled ORCHARDS weekly ARI	7.34	0.3, 3.68	0.0209
	Children attending school	0.92	−0.34, 0.18	0.5363
	Adults working outside house	0.83	−0.41, 0.03	0.0830

* Household density calculated as the number of household members per bedroom.

### 3.4 Additional Analysis of the March–June 2020 School Closure

**FIGURE 3:**
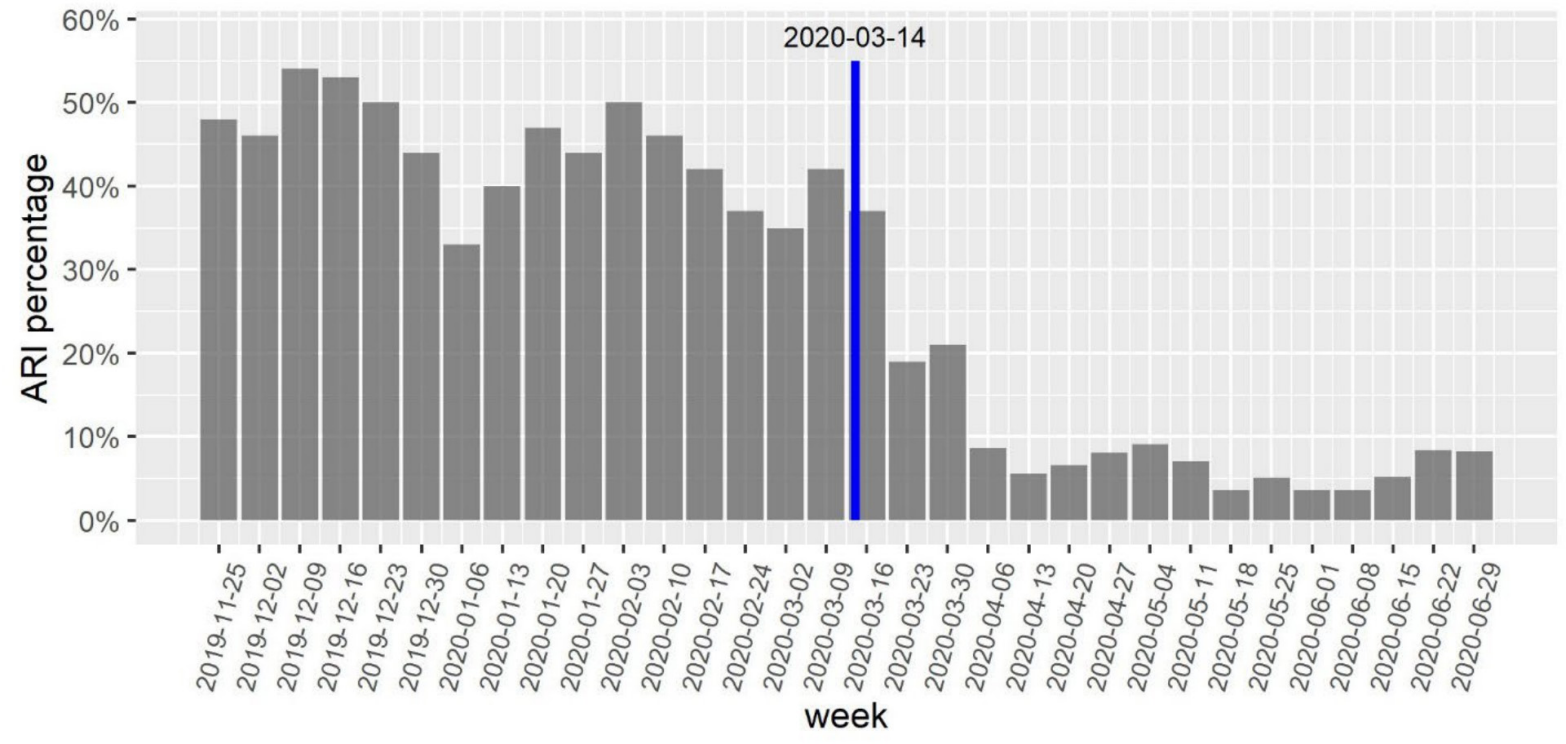
Weekly percent of households (ARI percentage) with at least one new acute respiratory infection in households within the Oregon School District (Dane County, WI, USA) in the 16 weeks before and after school closure due to COVID‐19 in March of 2020. A significant burden due to influenza was noted for Wisconsin in January and February 2020 (Wisconsin State Laboratory of Hygiene. Influenza Activity: 2019–2020 Influenza Activity. [cited 10 Oct 2022]. http://www.slh.wisc.edu/wcln‐surveillance/surveillance/virology‐surveillance/influenza‐activity/2019‐2020/).

During the 16 weeks prior to OSD school closure in March 2020, 44.4% of GROVES respondents indicated ≥ 1 household member with ARI during the previous week. In the 16 weeks following school closure, 10.2% indicated ≥ 1 household member with ARI during the previous week (Figure 3). Regression model slope/intercept interrupt was statistically significant (χ2 = 133, df = 2, *p* < 0.0001). Regression coefficients revealed both intercept and slope changes were statistically significant, with an intercept interrupt estimate indicating an immediate drop in household ARI odds corresponding to an OR of 0.51 (95% CI: 0.30–0.85) after school closure, and a slope change on the logit scale of −0.18 (95% CI: −0.19 to −0.05).

## 4 Discussion

From the earliest days of the COVID‐19 pandemic, an existing community‐based surveillance program using weekly surveys allowed us to measure fluctuating ARI activity in the OSD while incorporating the contextual elements of NPIs and school transitions at the beginning and end of the academic year, thus illustrating the impact of specific events on respiratory virus spread. We documented a significant reduction in community ARI risk with preemptive school closure in March 2020, with subsequent transition to fully online learning through June 2020. We also demonstrated significant increases in the trajectories of ARI risk corresponding with the end of two school years when K‐12 children presumably went from more restrictive to less restrictive NPI use, and at the beginning of two school years in which NPI employment was relaxed.

The statewide closure of public K‐12 schools in March of 2020 significantly impacted the Weekly % ARI reported among GROVES families when using a longer time window (±16 weeks), reflecting a similar finding based on Wisconsin rapid influenza testing in primary care clinics [21]. A previously published laboratory study of 670 archived samples collected between September 1, 2019, and June 30, 2020, documented only one household COVID‐19 cluster among ORCHARDS study participants, which occurred in March 2020 just before the schools were ordered to close [22]; this further confirmed very low ARI incidence observed in the GROVES cohort as reported here. Because peak influenza activity occurred in Wisconsin in January–February of 2020 [20], the natural decline in illness activity coincided with the COVID‐19 pandemic‐driven school closure, and the shorter time window used in the mixed effects logistic regression (±4 weeks) did not reveal a significant change in ARI risks trajectory. This finding is consistent with those from a systematic review of 34 studies assessing the effectiveness of NPIs in over 50 countries, which found school closure to be most effective for preventing the spread of SARS‐CoV‐2, followed by workplace closure, business closure, and public event bans [23].

### 4.1 Transition Event Associations

Transition events in this study included the return to school in September 2020, 2021, and 2022, the end of the school year in June 2020, 2021, and 2022, and lastly, two NPI‐related events in March 2020 and March 2022. Four of the eight transition events evaluated in this study were associated with an increase in ARI risk trajectory.

The return to school in September 2020 was marked by intense NPI utilization that included block scheduling for the morning and afternoon, mandatory face masking, physical distancing, and an optional hybrid model of schooling with a gradual return to in‐person activities. Approximately 25% of students opted for completely virtual learning. There was no significant change in ARI trajectory. This observational community‐based finding aligned well with the conclusions of a previously published modeling study that explored different scenarios for the reopening of US K‐12 schools for the school year 2020–2021 [24]. One study has proposed that children were not primary drivers of SARS‐CoV‐2 transmission due to reduced in‐school transmission when NPIs were in place [25]. Our findings lend credence to this assertion, implying the risk of spread was greater when school was out of session rather than in session when NPIs were implemented.

In contrast, return to school in September 2021 (mandatory face masking only) and September 2022 (no NPIs) was associated with significant increases in ARI trajectory. Thus, it stands to reason that more intensive NPIs employed at the beginning of the 2020–2021 school year when students were reintroduced to a common physical setting lowered the initial risk of ARI virus transmission.

The ends of the 2019–2020 and 2020–2021 school years in early June were associated with significant increases in ARI trajectories, while the end of the 2021–2022 school year was not. For the first two academic years, the beginning of the summer break marked a period of potentially lessened NPI utilization compared to the preceding school session, with potentially greater social mixing and heightened transmission risk as children left the school setting where structured NPIs prevented virus spread. In contrast, the transition to summer break in the 2021–2022 academic year did not present a significant change in NPI intensity, as the face mask requirement was rescinded after March 2022, and there were no NPIs in place when school ended in June 2022. The transition to optional face mask use in March of 2022 was associated with a slight but insignificant increase in ARI trajectory.

Of note is the virological composition of ARI cases within ORCHARDS during the study period [13, 26]. Influenza and most other virus detections disappeared with the closure of schools in March 2020. Low levels of rhinoviruses, SARS‐CoV‐2, and seasonal coronaviruses were detected during 2020–2021 when NPI use was maximal. When mandatory face masking remained as the only school‐based NPI during the 2021–2022 school year, virus diversity increased and was dominated by rhinoviruses (September through December) and SARS‐CoV‐2, coinciding with the SARS‐CoV‐2 omicron variant surge (January 2022) [13]. These observations align with other studies demonstrating reduced effectiveness of NPIs for preventing transmission of rhinoviruses in children [27, 28, 29, 30]. With the removal of all NPIs at the beginning of the 2022–2023 school year, an initial wave of rhinoviruses was followed by a swift influenza A resurgence in November 2022 [26].

### 4.2 Strengths and Limitations

This study has at least seven limitations. First, this was an observational study. The study team was not involved in setting the use and intensity of NPIs employed by the OSD, and there was no control group. Second, the findings are specific to a single school district in south central Wisconsin, thus limiting generalizability. Third, the assessment of ARI risk was confined to households that included children. Fourth, as surveys were distributed via email, participants needed access to the internet, which may have introduced demographic biases. Fifth, households were recruited from another local school‐based community surveillance study (ORCHARDS) and this process may have been susceptible to sampling bias. Accordingly, ARI risks may have been different for other households. Sixth, outside of weekly reporting rates, we did not measure adherence to protocol for GROVES. Finally, ARI cases were self‐reported, and we were unable to confirm the etiology for each individual participating in GROVES; however, we contextualized the results with those from the concomitant, laboratory‐supported ORCHARDS study.

This study also had notable strengths. First, we utilized an existing longitudinal cohort design for reporting of ARI that was in existence prior to the COVID‐19 pandemic. Second, we had impressive levels of adherence in reporting of > 94% over the 30 months covered by this study. Third, the long timespan of GROVES allowed capture of all NPI‐related transition events during the COVID‐19 pandemic in the OSD and surrounding community. Fourth, there was ongoing virological assessment within the community to contribute to this description of transition events. Finally, the community ARI trends reported by GROVES participants were similar to ARI incidence within ORCHARDS, enhancing the reliability of findings.

During the COVID‐19 pandemic, NPIs were deployed to reduce the spread of SARS‐CoV‐2 in‐school settings (32–34). This study demonstrated that school‐based NPIs may have additionally reduced respiratory virus transmission across the broader community. Informed evaluation of school transition events in the context of NPI utilization and retraction during an ongoing respiratory virus pandemic allows for a more meaningful interpretation of the associated effects and a deeper understanding of why these effects, if any, occurred. Because pandemics are inherently unpredictable, enhanced surveillance platforms, such as the one used in this study, should be prepositioned in a wide variety of locales to be able to further explore and understand the role schools and children have in SARS‐CoV‐2 and other respiratory virus transmission and the effectiveness of NPIs in preventing transmission of respiratory viruses in schools and surrounding communities.

## Author Contributions

**C He:** writing – original draft (lead). **MD Goss:** data curation (lead); writing – original draft (equal). **D Norton:** formal analysis (lead); writing – review and editing (equal); software (lead). **G Chen:** formal analysis (supporting); writing – review and editing (equal); software (supporting). **A Uzicanin:** writing – review and editing (equal). **JL Temte:** conceptualization (lead); methodology; supervision; writing – original draft (equal).

## Ethics Statement

All authors have reviewed and approved the final manuscript. The corresponding author has reviewed the Wiley Author Services *Best Practice Guidelines on Research Integrity and Publishing Ethics* and ensured the manuscript is in compliance with all guidelines.

## Conflicts of Interest

JLT is a member of the Advisory Board for Practice Update Primary Care. All other authors report no potential conflicts.

## Peer Review

The peer review history for this article is available at https://www.webofscience.com/api/gateway/wos/peer‐review/10.1111/irv.70139.

## Disclaimer

The findings and conclusions in this report are those of the authors and do not necessarily represent the official position of the Centers for Disease Control and Prevention.

## Supporting information


**Data S1.** Supporting information.

## Data Availability

The data that support the findings of this study are openly available in the Figshare data repository at https://figshare.com/articles/dataset/GROVES_regression_data/28583363?file=52949726, DOI: https://doi.org/10.6084/m9.figshare.28583363.v1.
