# Supplementary material for: Effects of K‐12 School District Nonpharmaceutical Interventions on Community‐Level Prevalence of Acute Respiratory Infection During the COVID‐19 Pandemic
Source: Influenza Other Respir Viruses. 2025 Jul 13;19(7):e70139. doi: 10.1111/irv.70139 (PMC12256271; doi:10.1111/irv.70139)
Supplement: Supplementary file 1 — Data S1. Supporting information. [file IRV-19-e70139-s001.pdf]

Weekly Survey

Please complete the Weekly Survey below.

Thank you!

Question	Response
Did you or any of your household members travel in the past week?	◯ Yes<br>◯ No
Where did you/they go?	__________________________________
Did any household member have a NEW influenza positive test in the past week?	◯ Yes<br>◯ No
How many household members (including yourself) had a NEW influenza positive test in the past week?	__________________________________
Did any household member have a NEW COVID-19 positive test in the past week?	◯ Yes<br>◯ No
How many household members (including yourself) had a NEW COVID-19 positive test in the past week?	__________________________________
Did anybody in your household have respiratory illness symptoms in the past 7 days (fever, runny/stuffy nose, cough, sore throat, sneezing)?	◯ Yes<br>◯ No
How many members of your household, including yourself, were sick?	__________________________________
Today's date	__________________________________

Participant #1

First Name	______________________
When did these symptoms start?	______________________
Severity of illness	◯ Mild<br>◯ Moderate<br>◯ Severe
Which symptoms has he/she experienced?	☐ Fever<br>☐ Chills<br>☐ Cough<br>☐ Runny Nose<br>☐ Stuffy Nose<br>☐ Sore throat<br>☐ Tiredness<br>☐ Body aches<br>☐ Headache<br>☐ Poor appetite<br>☐ Sneezing<br>☐ Shortness of breath<br>☐ Loss of taste and/or smell
Did he/she stay home from school or work?	◯ Yes<br>◯ No
How many days did he/she miss?	______________________
Has he/she seen a medical professional for this illness (virtual or in-person)?	◯ Yes<br>◯ No
Do you plan on seeing a medical professional for this illness?	◯ Yes, in-person<br>◯ Yes, virtual<br>◯ Yes<br>◯ No<br>◯ Maybe
What was the diagnosis?	______________________

Participant #2

First Name	______________________
When did these symptoms start?	______________________
Severity of illness	◯ Mild<br>◯ Moderate<br>◯ Severe
Which symptoms has he/she experienced?	☐ Fever<br>☐ Chills<br>☐ Cough<br>☐ Runny Nose<br>☐ Stuffy Nose<br>☐ Sore throat<br>☐ Tiredness<br>☐ Body aches<br>☐ Headache<br>☐ Poor appetite<br>☐ Sneezing<br>☐ Shortness of breath<br>☐ Loss of taste and/or smell
Did he/she stay home from school or work?	◯ Yes<br>◯ No
How many days did he/she miss?	______________________
Has he/she seen a medical professional for this illness (virtual or in-person)?	◯ Yes<br>◯ No
Do you plan on seeing a medical professional for this illness?	◯ Yes, in-person<br>◯ Yes, virtual<br>◯ Yes<br>◯ No<br>◯ Maybe
What was the diagnosis?	______________________

Participant #3

First Name

____

When did these symptoms start?

____

Severity of illness

- ◯ Mild
- ◯ Moderate
- ◯ Severe

Which symptoms has he/she experienced?

- ☐ Fever
- ☐ Chills
- ☐ Cough
- ☐ Runny Nose
- ☐ Stuffy Nose
- ☐ Sore throat
- ☐ Tiredness
- ☐ Body aches
- ☐ Headache
- ☐ Poor appetite
- ☐ Sneezing
- ☐ Shortness of breath
- ☐ Loss of taste and/or smell

Did he/she stay home from school or work?

- ◯ Yes
- ◯ No

How many days did he/she miss?

____

Has he/she seen a medical professional for this illness (virtual or in-person)?

- ◯ Yes
- ◯ No

Do you plan on seeing a medical professional for this illness?

- ◯ Yes, in-person
- ◯ Yes, virtual
- ◯ Yes
- ◯ No
- ◯ Maybe

What was the diagnosis?

____

Participant #4

First Name

____

When did these symptoms start?

____

Severity of illness

- ◯ Mild
- ◯ Moderate
- ◯ Severe

Which symptoms has he/she experienced?

- ☐ Fever
- ☐ Chills
- ☐ Cough
- ☐ Runny Nose
- ☐ Stuffy Nose
- ☐ Sore throat
- ☐ Tiredness
- ☐ Body aches
- ☐ Headache
- ☐ Poor appetite
- ☐ Sneezing
- ☐ Shortness of breath
- ☐ Loss of taste and/or smell

Did he/she stay home from school or work?

- ◯ Yes
- ◯ No

How many days did he/she miss?

____

Has he/she seen a medical professional for this illness (virtual or in-person)?

- ◯ Yes
- ◯ No

Do you plan on seeing a medical professional for this illness?

- ◯ Yes, in-person
- ◯ Yes, virtual
- ◯ Yes
- ◯ No
- ◯ Maybe

What was the diagnosis?

____

Participant #5

First Name

____

When did these symptoms start?

____

Severity of illness

- ◯ Mild
- ◯ Moderate
- ◯ Severe

Which symptoms has he/she experienced?

- ☐ Fever
- ☐ Chills
- ☐ Cough
- ☐ Runny Nose
- ☐ Stuffy Nose
- ☐ Sore throat
- ☐ Tiredness
- ☐ Body aches
- ☐ Headache
- ☐ Poor appetite
- ☐ Sneezing
- ☐ Shortness of breath
- ☐ Loss of taste and/or smell

Did he/she stay home from school or work?

- ◯ Yes
- ◯ No

How many days did he/she miss?

____

Has he/she seen a medical professional for this illness (virtual or in-person)?

- ◯ Yes
- ◯ No

Do you plan on seeing a medical professional for this illness?

- ◯ Yes, in-person
- ◯ Yes, virtual
- ◯ Yes
- ◯ No
- ◯ Maybe

What was the diagnosis?

____

Participant #6

First Name

____

When did these symptoms start?

____

Severity of illness

◯ Mild
◯ Moderate
◯ Severe

Which symptoms has he/she experienced?

☐ Fever
☐ Chills
☐ Cough
☐ Runny Nose
☐ Stuffy Nose
☐ Sore throat
☐ Tiredness
☐ Body aches
☐ Headache
☐ Poor appetite
☐ Sneezing
☐ Shortness of breath
☐ Loss of taste and/or smell

Did he/she stay home from school or work?

◯ Yes
◯ No

How many days did he/she miss?

____

Has he/she seen a medical professional for this illness (virtual or in-person)?

◯ Yes
◯ No

Do you plan on seeing a medical professional for this illness?

◯ Yes, in-person
◯ Yes, virtual
◯ Yes
◯ No
◯ Maybe

What was the diagnosis?

____

Participant #7

First Name

____

When did these symptoms start?

____

Severity of illness

- ◯ Mild
- ◯ Moderate
- ◯ Severe

Which symptoms has he/she experienced?

- ☐ Fever
- ☐ Chills
- ☐ Cough
- ☐ Runny Nose
- ☐ Stuffy Nose
- ☐ Sore throat
- ☐ Tiredness
- ☐ Body aches
- ☐ Headache
- ☐ Poor appetite
- ☐ Sneezing
- ☐ Shortness of breath
- ☐ Loss of taste and/or smell

Did he/she stay home from school or work?

- ◯ Yes
- ◯ No

How many days did he/she miss?

____

Has he/she seen a medical professional for this illness (virtual or in-person)?

- ◯ Yes
- ◯ No

Do you plan on seeing a medical professional for this illness?

- ◯ Yes, in-person
- ◯ Yes, virtual
- ◯ Yes
- ◯ No
- ◯ Maybe

What was the diagnosis?

____

Participant #8

First Name	__________________________________
When did these symptoms start?	__________________________________
Severity of illness	◯ Mild<br>◯ Moderate<br>◯ Severe
Which symptoms has he/she experienced?	☐ Fever<br>☐ Chills<br>☐ Cough<br>☐ Runny Nose<br>☐ Stuffy Nose<br>☐ Sore throat<br>☐ Tiredness<br>☐ Body aches<br>☐ Headache<br>☐ Poor appetite<br>☐ Sneezing<br>☐ Shortness of breath<br>☐ Loss of taste and/or smell
Did he/she stay home from school or work?	◯ Yes<br>◯ No
How many days did he/she miss?	__________________________________
Has he/she seen a medical professional for this illness (virtual or in-person)?	◯ Yes<br>◯ No
Do you plan on seeing a medical professional for this illness?	◯ Yes, in-person<br>◯ Yes, virtual<br>◯ Yes<br>◯ No<br>◯ Maybe
What was the diagnosis?	__________________________________

Thank you for completing this survey.
